# The Mechanism by Which Estrogen Level Affects Knee Osteoarthritis Pain in Perimenopause and Non-Pharmacological Measures

**DOI:** 10.3390/ijms26062391

**Published:** 2025-03-07

**Authors:** Huiying Zhao, Fan Yu, Wei Wu

**Affiliations:** 1 School of Exercise and Health, Shanghai University of Sports, Shanghai 200438, China; zhaohy1237@163.com (H.Z.); 13734934551@163.com (F.Y.); 2 School of Athletic Performance, Shanghai University of Sports, Shanghai 200438, China

**Keywords:** perimenopause, estrogen, knee osteoarthritis, non-pharmacological measure

## Abstract

Perimenopausal women have fluctuating estrogen levels, which often trigger a range of symptoms of perimenopausal syndromes as estrogen levels decrease. Changes in perimenopausal estrogen levels are closely related to pain in knee osteoarthritis (KOA), which has long been a research area of great interest in perimenopausal women. In recent years, it has been found that perimenopausal estrogen levels have an important role in KOA pain, namely, that estrogen can affect KOA pain through the regulation of inflammatory responses, inhibition of cellular senescence and apoptosis, and modulation of neurotransmitters, which may provide new ideas for KOA treatment. This study aims to describe the mechanism of estrogen level on knee osteoarthritis pain in perimenopause and related non-pharmacological measures, such as physical therapy, physical factor therapy, traditional Chinese medicine, and diet, which can provide a reference for the study and treatment of pain in perimenopausal women with KOA.

## 1. Introduction

Perimenopause is the transitional phase surrounding a woman’s menopause, marking a decline in ovarian function. This phase lasts until one year after her final menstrual period [1]. As women enter the perimenopausal period, they experience a range of physiological and psychological changes, primarily due to significant fluctuations in estrogen levels [2]. Estrogen plays a crucial role in the health and quality of life of perimenopausal women, and as ovarian function declines, estrogen production significantly decreases and becomes more erratic [3]. This decline is not gradual; instead, it occurs sharply, often leading to a variety of symptoms associated with perimenopause [4]. These symptoms can include hot flashes, dryness, and autonomic dysregulation, which may be accompanied by dizziness, insomnia, and heart palpitations [5].

Changes in estrogen levels among perimenopausal women are closely linked to the onset of knee osteoarthritis (KOA), and decreased estrogen levels can trigger pain associated with KOA [6]. Estrogen plays a protective role in maintaining bone and cartilage health, and reduced levels can lead to the degeneration of articular cartilage and contribute to the development of arthritis, worsening KOA [7]. The onset of KOA is becoming increasingly common, with epidemiological data indicating a higher prevalence among women and obese individuals. Women tend to experience the onset of KOA at a younger average age compared to men, and the severity of their condition is often greater. Additionally, postmenopausal women make up a larger proportion of KOA cases [8].

In this review, we systematically reviewed the related mechanisms of pain production in KOA and further summarized the potential mechanisms by which changes in estrogen levels affect KOA pain. In addition, we discussed related non-pharmacological measures that can be performed by perimenopausal women with KOA, aiming to better help reductions in KOA pain.

## 2. KOA Pain

The mechanism of KOA pain is multifactorial and complex, including tissue damage, cell senescence and apoptosis, nerve sensitization, biomechanics, and psychology. Among them, the most common causes of KOA pain mainly involve three aspects: tissue damage, cell senescence and apoptosis, nerve sensitization.

### 2.1. Concept of KOA

KOA is a chronic degenerative disease marked by the degeneration of articular cartilage and the formation of joint osteophytes. This condition leads to knee pain and limits activities, significantly impacting the patient’s quality of life [9]. The causes of KOA include factors such as age, gender, weight, genetics, injuries, and overuse [10]. In the early stages of the disease, individuals experience intermittent pain that, as the disease progresses to the middle stages, develops into persistent dull pain occurring more frequently. In the late stages, this dull pain can become a constant issue, accompanied by intense, sharp pain that arises intermittently [11]. KOA cannot be reversed; it is characterized by pain, limited mobility, and bone spurs in the joints [12]. The diagnosis of KOA typically involves a review of the patient’s history, a physical examination, and imaging studies. Common symptoms include joint pain, swelling, stiffness, and decreased range of motion [13].

### 2.2. Mechanisms of KOA Pain

#### 2.2.1. Tissue Damage

The pain associated with KOA is linked to damage in various tissues, including the articular cartilage, subchondral bone, synovium, infrapatellar fat pad, meniscus, and anterior cruciate ligament [14]. This tissue damage triggers the release of pro-inflammatory factors, such as interleukin-1β (IL-1β), tumor necrosis factor-α (TNF-α), and interleukin-6 (IL-6), leading to an inflammatory response [15]. When tissue injury occurs, the local structure and mechanical properties at the site of the injury are altered, which can further stimulate pain receptors and contribute to the sensation of pain [16]. The two primary causes of KOA are degenerative damage to articular cartilage and subchondral bone [17]. Articular cartilage is believed to be one of the first areas affected by the disease [18]. Normally, articular cartilage does not contain blood vessels or nerves, which is why it does not produce pain [19]; however, following degenerative damage to the cartilage, small blood vessels begin to form at the injury site. Chondrocytes in the damaged area release pro-angiogenic factors, nerve growth factor (NGF), and neuropeptides, which stimulate pain sensations [20]. Damage to the subchondral bone involves multiple processes, including subchondral bone remodeling, damage to the bone marrow, and the formation of osteophytosis. This damage often leads to the exposure of the subchondral bone plate, which causes an increase in blood vessels and fibrous tissue beneath the bone’s surface. Consequently, osteophytosis gradually forms, leading to pain [21]. Tissue damage can lead to localized defects in the subchondral bone, which increases stress on the cartilage [22]. This added pressure may result in the breaking of trabecular bone, activating pain receptors in both the subchondral bone and bone marrow [23]. Consequently, this process stimulates the growth of blood vessels and new bone formation, while pro-inflammatory factors also contribute to the sensation of pain [24].

#### 2.2.2. Cell Senescence and Apoptosis

There is a link between chondrocyte senescence and the development of KOA. The senescence-associated secretory phenotype (SASP) and extracellular vesicles (EVs) produced by senescent chondrocytes affect surrounding cells and worsen the progression of KOA [25]. EVs in KOA synovial fluid contain pro-inflammatory factors that activate synoviocytes and chondrocytes, leading to inflammation and cellular senescence [26]. SASP is an umbrella term for pro-inflammatory cytokines, chemokines, and matrix metalloproteinases involved in joint tissue destruction, which can exacerbate the inflammatory response by promoting the secretion of inflammatory factors and mediators, such as IL-β, TNF-α, IL-6, and matrix metalloproteinases (MMPs), stimulating neighboring cellular senescence and triggering pain [27]. MMPs secreted by the SASP degrade the extracellular matrix of cartilage, leading to structural and functional impairment of cartilage, which is one of the main factors causing pain in KOA [28]. Apoptosis also plays a key role in the pathogenesis of KOA [29]. Vascular endothelial growth factor (VEGF), which is an SASP, is an essential cytokine for osteogenesis in cartilage [30]. VEGF, which promotes the formation of osteoclasts, increases the expression of MMP-2 and MMP-9 and accelerates chondrocyte apoptosis [31]. Apoptosis is not just a form of cell death; it may also indirectly or directly trigger pain by affecting nervous system function and signaling [32].

#### 2.2.3. Nerve Sensitization

**Figure 1:**
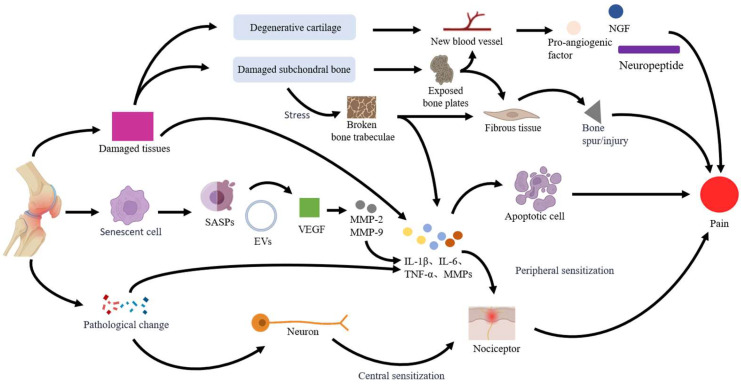
Pain in KOA primarily results from tissue damage, cellular senescence and apoptosis, and nerve sensitization. Inflammation will occur at the site of tissue injury, and it will also cause the proliferation of new small blood vessels and fibrous tissues at the site of injury, forming osteophytes and causing pain. Senescent and apoptotic cells release pro-inflammatory factors, exacerbating inflammation and increasing pain. This inflammatory process lowers the pain threshold and leads to peripheral sensitization, ultimately heightening sensitivity to pain signals and intensifying overall pain over time.

There are changes in nerve function associated with KOA, including both peripheral and central nerve sensitization, which are closely related to the development of pain. Nerve sensitization refers to the heightened sensitivity of nerves to stimuli, leading to lower pain thresholds and an increased perception of pain. This sensitization can be triggered by inflammation, injury, or ongoing harmful stimuli, and it may contribute to the chronicity and amplification of pain [33]. The wear of articular cartilage and osteomalacia resulting from KOA change the intra-articular environment, leading to an elevated release of inflammatory factors and NGF. These substances activate injury receptors, particularly free nerve endings located in the synovium, periosteum, and tendons, which in turn trigger pain signals [34]. Prolonged inflammatory stimulation lowers the excitatory threshold of injury receptors, leading to an exaggerated response to normal stimuli and resulting in peripheral sensitization and increased pain [35]. Prolonged stimulation of peripheral pain can lead to changes in the central nervous system, resulting in central sensitization. When central sensitization occurs, neurons in the spinal cord and brain change how they process and perceive pain signals. This alteration results in increased sensitivity to pain and a broader range of pain experiences [36]. Chronic joint pain in patients with KOA can lead to alterations in gray matter volume within the brain. Notably, there is a reduction in gray matter in the insula, parietal lobe, and prefrontal cortex, while an increase is observed in the frontal lobe, amygdala, and nucleus accumbens. These changes influence pain-related brain regions, amplifying the perception and experience of pain (Figure 1) [37].

Tissue damage, cell senescence and apoptosis, and nerve sensitization are the most common causes of KOA pain, with changes in estrogen levels associated with these three factors [38,39,40].

## 3. Perimenopausal Estrogen Level and KOA Pain

### 3.1. Changes in Estrogen Levels of Perimenopausal Women

The concept of the perimenopausal woman was first introduced in 1994 at the Conference on Advances in Menopause Research in the 1990s, convened by the WHO Special Planning Committee on Human Reproduction. The perimenopausal period is usually categorized as the early menopausal transition period, the late menopausal transition period, and the 12 months after menopause [41]. During the early menopausal transition, menstrual cycles become irregular and frequent. Ovarian feedback to the pituitary gonadotropins, follicle-stimulating hormone (FSH), and luteinizing hormone (LH) is reduced, leading to shorter follicular phases, fewer ovulations, and decreased progesterone production [42]. Estrogen levels show a fluctuating, gradual decline. Changes in FSH levels were used as a reference to determine when a woman in late menopausal transition officially entered menopause [43]: when FSH levels reached or exceeded 25 U/L after the last menstrual period and the length of the menstrual cycle was 60 days or more [44]. During this phase, estrogen levels fluctuate and gradually decline. The year following the final menstruation represents the +1a stage of early late menopause, which signals the start of official menopause. The +1b phase commences one year later, during which hormone levels continue to fluctuate. As we transition into the +1c phase, FSH levels rise steadily, while estradiol levels remain low [45]. After menopause, estrogen shows more stable low levels [46]. The overall change in estrogen levels in perimenopausal women is a gradual decline from fluctuating and relatively high levels to stable low levels after menopause.

**Figure 2:**
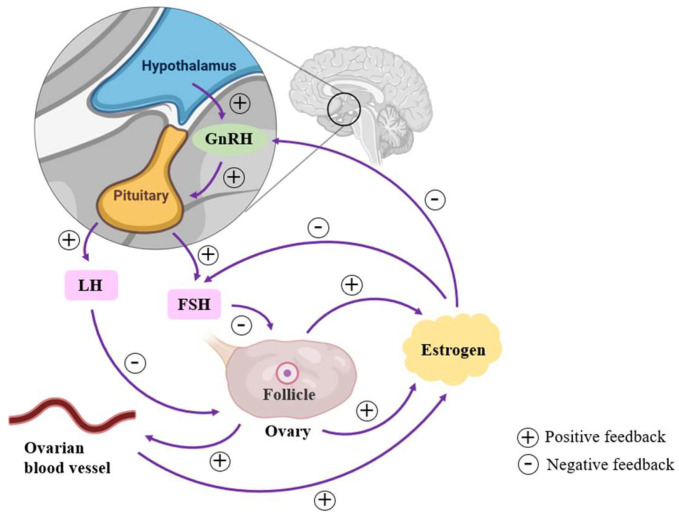
In perimenopausal women, ovarian function decline has a direct or indirect impact on follicle, ovarian blood vessel function, and estrogen levels. The decline in estrogen levels due to ovarian function decline affects the HPOA. The negative feedback of estrogen is diminished, allowing the hypothalamus and pituitary to secrete more GnRH, FSH, and LH. FSH and LH have negative feedback on ovarian function.

The central change in perimenopausal women is the gradual decline of ovarian function, which can directly or indirectly affect estrogen levels [47]. The number of follicles in the ovaries declines with age [48], and during perimenopause, this number decreases significantly and in some cases they fail to mature, leading to a drop in estrogen levels. Decreased ovarian function leads to a decrease in the function of its internal blood vessels, resulting in a decreased blood supply within the ovaries. Moreover, inadequate blood supply affects follicle development and estrogen synthesis, leading to decreased estrogen levels [49]. The ovaries secrete estrogen and progesterone according to instructions from the pituitary. The hypothalamic–pituitary–ovarian axis (HPOA) is a key neuroendocrine system in the female reproductive system. The hypothalamus is the starting point and secretes gonadotropin-releasing hormone (GnRH). GnRH passes through the pituitary portal system to the pituitary, which receives instructions from the hypothalamus to secrete FSH and LH, which act on the ovaries to produce estrogen [50]. Under normal conditions, elevated estrogen levels exert negative feedback on the hypothalamus and pituitary, decreasing the secretion of GnRH, FSH, and LH [51]. During perimenopause, when ovarian function declines, resulting in a significant decrease in estrogen levels, the negative feedback is diminished, allowing the hypothalamus and pituitary to perceive estrogen insufficiency and thus increase GnRH, FSH, and LH secretion. There is some negative feedback between them and ovarian function (Figure 2).

### 3.2. Mechanisms of Estrogen Levels on KOA Pain

Estrogen is a steroid hormone produced mainly by the ovaries, including estrone, estradiol, estriol, and estosterol, and is essential for women’s reproductive and endocrine health [52]. During the perimenopausal period, there is a fluctuating decline in estrogen levels in women, and the incidence of KOA is higher during this period, which is closely related to changes in estrogen levels [53]. Estrogen can influence the inflammatory response and disease progression in perimenopausal women with KOA through the regulation of inflammatory responses, inhibition of cellular senescence and apoptosis, and modulation of neurotransmitters.

#### 3.2.1. Regulation of Inflammatory Response

Pain in KOA commonly arises from tissue damage that triggers inflammation. This inflammation releases various mediators, such as IL-1β, TNF-α, and prostaglandin E2, which directly activate pain receptors in the joints, leading to discomfort [54]. High expression of inflammatory factors inhibits the biosynthesis of extracellular matrix proteins in chondrocytes and promotes the expression of degradative enzymes such as collagenase, gelatinase, and matrix lysin in MMPs, leading to the destruction of articular cartilage and the initiation of pain [55]. Studies have shown that the level of IL-1β is higher in the joint fluid of patients with KOA than in normal subjects, and that KOA is more severe as IL-1β increases [56]. TNF-α stimulates the production of MMPs and other degrading enzymes by chondrocytes, accelerating the degradation process of articular cartilage and exacerbating the development of KOA [57].

Estrogen has a certain anti-inflammatory effect, which can inhibit the release of inflammatory factors such as TNF-α and IL-1β and reduce the pain caused by inflammatory response [58]. A decrease in estrogen leads to increased destruction of articular cartilage and subchondral bone and an inability to regulate the balance between osteoblasts and osteoclasts, resulting in cartilage erosion, destruction, and the formation of bony encumbrances, which trigger pain [59]. As estrogen levels decline, the rate of subchondral bone conversion increases significantly, with frequent remodeling at the site of injury, further sclerosis of the subchondral bone, and altered biomechanical status at the joint, further exacerbating the progression of KOA [60]. At the same time, estrogen levels are directly proportional to the levels of estrogen in the joint fluid, and reduced estrogen in the joint fluid leads to reduced inhibition of cartilage-destroying inflammatory factors in the joints. In an animal model study, it was found that after ovariectomy, the level of estrogen in the joint fluid of mice decreased, leading to increased damage to articular cartilage and severe arthritis [61].

#### 3.2.2. Inhibition of Cellular Senescence and Apoptosis

Cellular senescence and apoptosis are the key factors in the development of KOA and a major contributor to pain [62]. With age, senescent chondrocytes in the joints gradually lose their ability to synthesize and repair matrix members, leading to degradation of the cartilage matrix and destruction of cartilage structure, which ultimately triggers apoptosis and thus accelerates the development of KOA [63]. At the same time, inflammatory factors and cellular debris released during apoptosis further exacerbate chondrocyte senescence [64].

Estrogen plays a crucial role in regulating cell senescence and apoptosis [39] and influences chondrocyte proliferation, reduces oxidative stress, inhibits chondrocyte apoptosis, and modulates the expression of apoptosis-related signaling pathways and proteins. By activating estrogen receptors (ERs), estrogen promotes chondrocyte proliferation, which helps delay cell senescence and apoptosis. The estrogen receptor alpha (ERα) is particularly important in this process, serving as a key regulator of cell senescence and apoptosis. Stressors, such as mechanical overload, cause a decrease in ERα levels, which promotes chondrocyte senescence [65]. ERα helps reduce oxidative stress and lowers the excessive production of reactive oxygen species (ROS). It inhibits the expression of markers associated with cellular senescence, delays the aging process of cells and apoptosis, and alleviates the severity of KOA by regulating apoptosis-related signaling pathways, such as PI3K/Akt/mTOR and MAPK [66]. ERα plays an important role in cell proliferation, and ERα is expressed in metabolically active tissues and regulates the transcription of metabolic genes, thereby affecting the proliferation and regeneration of osteoblasts, chondrocytes, and muscle cells [67]. In addition, ERα can inhibit chondrocyte apoptosis by affecting the expression of apoptosis-related proteins, such as the B-cell lymphoma-2 (Bcl-2) gene and Bcl-2-associated X protein (BAX). It was found that ERα could increase the expression of Bcl-2 and decrease the expression of Bax, thus inhibiting chondrocyte apoptosis [68]. G protein-coupled estrogen receptor (GPER) also plays a key role in regulating mechanical stress-mediated chondrocyte apoptosis. The expression of GPER in cartilage was found to be negatively correlated with cartilage degeneration, and the activation of GPER could reduce the apoptosis of chondrocytes induced by mechanical stress, alleviate the course of KOA, and reduce the pain caused by KOA [69].

#### 3.2.3. Modulation of Neurotransmitters

KOA pain arises from the interaction between the central and peripheral nervous systems. Patients experiencing long-term KOA often have a diminished pain threshold due to nerve sensitization, which heightens their pain sensitivity. Estrogen plays a crucial role in regulating pain perception by influencing the release and metabolism of neurotransmitters.

**Figure 3:**
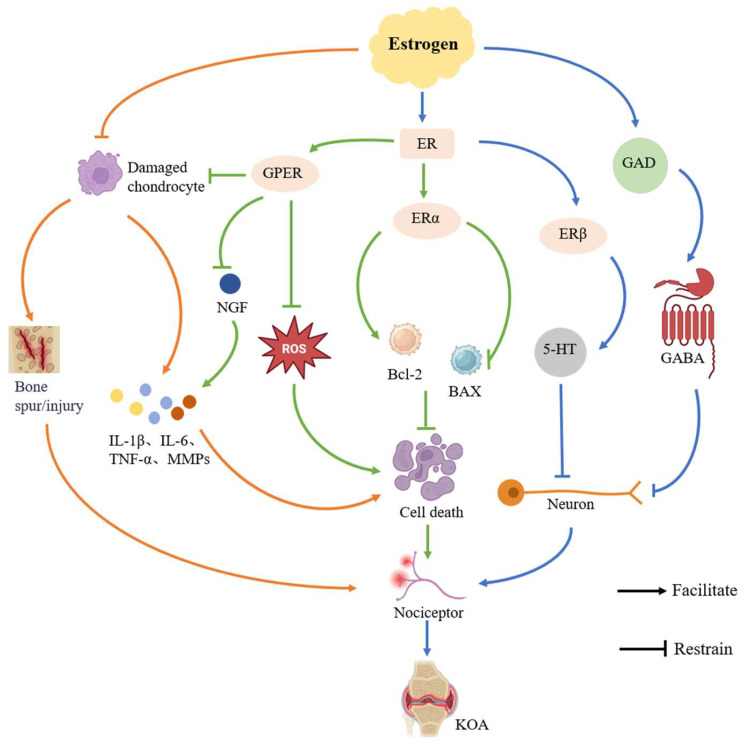
A drop in estrogen can cause KOA pain. Estrogen can regulate the inflammatory response, inhibit the injured chondrocytes, prevent the formation of TNF-α, IL-1β and other inflammatory factors and osteophytes, reduce cell aging, reduce the stimulation of nociceptors, and reduce KOA pain. Estrogen can activate ER, produce GPER, inhibit the apoptosis of chondrocytes, reduce oxidative stress, decrease the overproduction of ROS and NGF, increase the expression of Bcl2, reduce the expression of BAX, reduce inflammatory response, slow down cell death, reduce the stimulation of nociceptors, and relieve KOA pain. Estrogen can regulate relevant neurotransmitters to raise pain thresholds and reduce pain. Estrogen, through ER, regulates related neurotransmitters such as GAD, GABA, and 5-HT, inhibits neuronal activity, reduces stimulation of nociceptors, and reduces KOA pain.

Estrogen modulates pain in the central nervous system by interacting with its receptors, such as ERα and estrogen receptor beta (ERβ). ERα co-localizes with proenkephalin precursor mRNA in the spinal cord and increases spinal enkephalin levels upon activation, leading to analgesic effects. In contrast, ERβ mainly exerts analgesic effects by enhancing the downstream inhibitory pathway of 5-hydroxytryptamine (5-HT) [70]. 5-HT is an important neurotransmitter that is closely related to the perception of pain [71]. Estrogen can influence the synthesis and release of 5-HT, modulating the activity of brain regions associated with pain processing, such as the amygdala, thalamus, and anterior cingulate cortex, which in turn affects pain perception [72]. Estrogen can further increase the concentration and metabolism of 5-HT by modulating neuronal excitability, affecting its role in the nervous system and influencing pain thresholds [73]. It has been shown that estrogen can affect pain perception by modulating the activity of 5-HTergic neurons in the hypothalamus-dorsal nucleus of the middle suture loop [74]. Gamma-aminobutyric acid (GABA), another important neurotransmitter, is catalyzed by glutamate decarboxylase (GAD) to produce glutamate, which plays a key role in the regulation of pain sensation [75]. Studies have shown that estrogen can affect GABA synthesis, inhibit neuronal excitability, affect amygdala activity, modulate pain-related emotional responses, and reduce pain signaling by regulating GAD activity or expression through its receptors [76]. Elevated estrogen levels significantly enhance GABAergic neurotransmission, leading to a notable increase in pain tolerance [77]. Estrogen modulates injury receptor activity and pain signaling in the peripheral nervous system by regulating the expression of specific ion channels, such as TRPV1 and P2X3. Additionally, it influences NGF expression through the activation of GPER, impacting injury receptor sensitivity and neuronal function, which ultimately contributes to pain reduction (Figure 3) [78].

## 4. Non-Pharmacological Measures in Perimenopausal Women with KOA

Currently, pharmacologic treatments, such as analgesics or nonsteroidal anti-inflammatory drugs, are the most common clinical treatments for KOA pain in perimenopausal women [79]. However, due to the complex mechanism of KOA pain in perimenopausal women, medication can only alleviate pain symptoms; there is no cure. Meanwhile, long-term use of medication will inevitably produce adverse effects, which may increase liver and kidney toxicity, cardiovascular disease risk, and gastrointestinal disorders [80].

In recent years, with the increased attention paid to the treatment of women with perimenopausal KOA and the introduction of various international guidelines, non-pharmacological measures have been strongly recommended, often with better results. Non-pharmacological measures are advantageous due to their high safety and cost effectiveness, and they can effectively avoid the side effects associated with hormone therapy while also alleviating financial burdens.

### 4.1. Physical Therapy

As estrogen levels decrease, perimenopausal women with KOA experience a series of physical and mental problems such as autonomic dysfunction, hot flashes, and insomnia, in addition to KOA-induced pain [81]. Physical therapy (PT), as a non-pharmacological measure, does not produce hormone-related side effects and is useful for the prevention and relief of pain in perimenopausal women with KOA [82]. PT mainly involves aerobic exercises, resistance training, and mind–body exercises.

#### 4.1.1. Aerobic Exercise

Aerobic exercise (AE), as a good stimulus, can cause the central β-endorphin content to increase, such that the hypothalamus–pituitary–ovary axis produces adaptive effects, which in turn improves ovarian function, increases estrogen secretion, and relieves the pain produced by osteoarthritis of the knee [83]. Perimenopausal women who engage in AE for 40 min, including a 5 min warm-up and a 5 min cool-down, five times a week can significantly alleviate knee osteoarthritis pain and its related dysfunction. Walking or cycling are effective, and the intensity of training should be maintained at a moderate level, specifically 50% to 70% of the maximum heart rate, which is calculated as 220 minus the individual’s age [84]. Decreased estrogen levels in perimenopausal women are accompanied by autonomic dysfunction and decreased heart rate variability (HRV) levels, which may increase the risk of cardiovascular disease [85]. Engaging in appropriate moderate-intensity AE can improve the body’s cardiorespiratory function, enhance the activity of both sympathetic and parasympathetic nerves, and help prevent and reduce the risk of cardiovascular disease [86].

#### 4.1.2. Resistance Training

Resistance training (RT) can enhance muscle strength and improve body function. Medium- to high-intensity RT for 60 min, two to three times a week, can effectively regulate hormone levels in perimenopausal women [87]. The Chinese KOA Rehabilitation Treatment Guidelines for 2023 state that patients with KOA should engage in plyometric and RT, which can be targeted to the quadriceps muscles [88]. RT involves a range of exercises, including squats, push-ups, and hard pulls, covering the major muscle groups of the entire body. According to certain research, RT not only raised estradiol levels but also markedly decreased high-sensitivity C-reactive protein levels. This suggests that RT may have an anti-inflammatory effect and can lessen the production and release of inflammatory factors, which in turn may lessen the pain associated with osteoarthritis in the knees in perimenopausal women [89]. RT has a significant positive effect on the regulation of hormone levels, skeletal muscle strength, and body composition in perimenopausal women, effectively improving the quality of life of perimenopausal women and reducing the pain associated with KOA.

#### 4.1.3. Mind–Body Exercise

Mind–Body exercise (MBE) is a form of exercise that combines attention and physical movement to enhance mind–body coordination and awareness [90]. Perimenopausal KOA women can use Tai Chi, Baduanjin, and Qigong to improve KOA pain according to their preferences. MBE may effectively alleviate pain, irritation, and hot flashes, balance sympathetic and parasympathetic nerves, control estrogen levels, and enhance the physical and emotional well-being of perimenopausal KOA women [91]. MBE, like Baduanjin exercises or traditional Qigong, can effectively lower the production of inflammatory substances and has a beneficial regulatory effect on women’s estrogen levels [92]. Tai Chi ball exercise has been shown in a study to dramatically raise estrogen levels in perimenopausal women, improve muscle strength and bone metabolism indicators, alleviate symptoms of perimenopausal women, lessen KOA pain, and prevent osteoporosis, which is likely to develop later [93]. A 12-week randomized controlled trial reveals that practicing Qigong improves the severity of perimenopausal symptoms and health-related quality of life [94]. According to the literature and related studies, it is recommended that perimenopausal women with osteoarthritis of the knee undergo MBE 3–4 times per week for about 60 min each time, which can effectively regulate estrogen levels and alleviate pain caused by KOA in perimenopausal women with KOA [95].

Additionally, perimenopausal women with KOA are capable of performing some neuromuscular, proprioceptive, balancing, and joint mobility training. These are great exercises for enhancing neuromuscular control and functional joint stability, maximizing the biomechanical efficiency of the lower extremities, improving balance, lowering the risk of falls, and minimizing common injuries [96].

### 4.2. Physical Factor Therapy

Physical factor therapy (PFT) is a conventional approach to relieve pain, reduce inflammation, and improve joint function through a variety of physical means. Low-Intensity Pulsed Ultrasound (LIPUS), Transcutaneous Electrical Nerve Stimulation (TENS), and traditional Chinese medicine (TCM) are often used as adjunctive treatments.

#### 4.2.1. Low-Intensity Pulsed Ultrasound

LIPUS transmits high-frequency pressure waves to the tissues of the articular cartilage, joint capsule, and synovium, where the sound waves propagate through the medium causing vibrations and collisions and producing effects [97]. Previous research has demonstrated that LIPUS plays a part in soft tissue regeneration and repair as well as anti-inflammation, which can successfully prevent the production of inflammatory components [98]. LIPUS was also found to promote the expression of growth differentiation factor 9 (GDF9) and bone morphogenetic protein 15 (BMP15) in rat ovaries. GDF9 and BMP15 are glycoproteins secreted by oocytes and are important oocyte autocrine factors. By regulating the expression of these two factors, LIPUS plays a protective and restorative role in ovarian function, which in turn regulates estrogen levels and can reduce pain in perimenopausal women with KOA [99]. In addition, LIPUS can change the pH of tissue fluid, reduce the excitability of sensory nerves, and increase the pain threshold, while inhibiting the release of neurotransmitters to achieve the analgesic effect. The study showed that after LIPUS treatment, the levels of IL-1β, IL-6, and TNF-α were significantly reduced, and the therapeutic effect could be maintained for at least 3 months [100]. Based on the study, it is recommended that perimenopausal women with KOA undergo 3–5 LIPUS treatments per week, with each treatment lasting approximately 20 min, with the exact frequency and duration of treatments being adjusted according to the specific situation and response to treatment [101].

#### 4.2.2. Transcutaneous Electrical Nerve Stimulation

TENS activates peripheral nerve fibers by applying a current of specific parameters to the skin surface and reduces injury perception signals, thus providing relief for acute and chronic pain [102]. TENS is frequently used in clinical practice because it is affordable, safe, and non-invasive. In addition to common acupoints such as Zusanli, Yanglingquan, and Yinlingquan, TENS can be applied to specific acupoints such as Guanyuan, Sanyinjiao, and Tianshu. Doing these can significantly raise a woman’s serum estradiol level and, to some extent, alleviate KOA pain [103]. One study found that TENS therapy produced similar results to electric acupuncture (EA) for perimenopause-related symptoms and also relieved pain in perimenopausal women with KOA [104]. TENS, as an effective modality in the conservative treatment of KOA, is more significant in improving KOA pain, joint mobility, and quality of life, and the analgesic effect of high-frequency TENS is better than that of low-frequency TENS. High-frequency TENS (80–110 Hz) can stimulate primary sensory afferent skin fibers, activate inhibitory interneurons in the dorsal horn of the spinal cord so that pain will not be transmitted to higher centers, and reduce cortisol levels, increase endorphins, and exert analgesic effects [105]. One study compared the effectiveness of EA and TENS for KOA pain control and found that high-frequency TENS was more suitable for treating pain associated with KOA, with EA as a second choice [106]. Based on relevant studies, five weekly treatments of approximately 20–40 min each are recommended for perimenopausal women with KOA, with the exact frequency and duration of treatments to be adjusted according to the specific situation and response to treatment [107].

### 4.3. Traditional Chinese Medicine

Acupuncture and moxibustion, as common traditional Chinese medicine (TCM) therapies, are useful in relieving KOA pain. Dubi, Yanglingquan, Yinlingquan, and Zusanli are widely used as common acupuncture points for KOA and can alleviate pain and lower blood levels of inflammatory markers like IL-1β and IL-6 [108,109]. Furthermore, in perimenopausal women with KOA, acupuncture locations such as Sanyinjiao and Taixi can increase estrogen levels, particularly E2, and reduce KOA symptoms [110]. Acupuncture can affect the neuroendocrine regulatory mechanism of the hypothalamic–pituitary–ovarian axis by stimulating specific acupoints and promoting the secretion of estrogen by the ovaries, thus increasing the level of estrogen in the serum and alleviating pain and perimenopausal-related symptoms [111]. Moxibustion, another TCM, acts on specific acupuncture points through warm stimulation and the medicinal effects of mugwort. Moxibustion applied to the Guanyuan and Sanyinjiao points can significantly elevate estrogen levels, promote estrogen receptor expression, and relieve KOA pain [112]. This study demonstrated that moxibustion could raise estrogen levels and boost the expression of estrogen receptors in ovarian tissues through the PI3K/AKT signaling pathway, and it was discovered that rats’ E2 serum levels were considerably higher following moxibustion than in the control group [113]. According to relevant studies, it is recommended that the length of each acupuncture treatment be limited to 20–30 min for perimenopausal women with KOA, with three to four acupuncture sessions per week [114]. The duration of moxibustion treatment is 15–20 min per session, three to five times per week, and the specific frequency and duration of treatment should be adjusted according to the specific situation and response to treatment [115].

### 4.4. Diet

Phytoestrogens are naturally occurring plant compounds with estrogenic activity that are found in a variety of foods. The chemical structure of phytoestrogens is similar to that of human estrogens, and they can exert estrogen-like effects [116]. Studies have demonstrated that the potential health advantages of phytoestrogens can effectively alleviate several menopausal discomforts, including mood swings, hot flashes, and night sweats [117]. In addition, phytoestrogens can significantly improve bone health and provide some cardiovascular protection [118]. Soybeans, soy products, flaxseeds, and chickpeas are examples of phytoestrogens that perimenopausal women with KOA can include in their regular diet to help control estrogen levels and alleviate the pain sensations associated with the condition.

One significant phytoestrogen found in soybeans and soy products is soy isoflavones [119]. Because they bind to the ER and activate ER-mediated signaling pathways, isoflavones, which share structural similarities with 17-beta-estradiol, can have effects comparable to those of estrogen [120]. Estrogen can also be controlled by isoflavones. Isoflavones have estrogen-related effects when there is not enough estrogen, and they serve as an estrogen antagonist when there is too much estrogen by blocking the ER that binds estrogen [121]. Additionally, it has been demonstrated that isoflavones improve bone health by controlling signaling pathways in bone tissue cells, which increases bone density and improves some of the biomechanical characteristics of bone, while also inhibiting osteoclastogenesis and activity [122]. The primary ingredient in flaxseed is lignans, which have a structure similar to that of estrogen and can be coupled with ER to control the body’s levels of estrogen. According to a study evaluating the effects of flaxseed and soy on estrogen metabolism in postmenopausal women, flaxseed can considerably regulate estrogen levels and may even have a greater impact than soy [123]. Chickpeas are rich in isoflavones, which contain isoflavonoids, such as Biochanin-A (BCA), that have estrogen-like effects and can alleviate perimenopause-related symptoms [124]. BCA has beneficial effects on bone health by inhibiting adipocyte development and promoting osteoblast differentiation through ER-related signaling [125]. Vitamin D can affect estrogen synthesis by regulating aromatase activity, and appropriate supplementation can increase estrogen levels and affect bone health [126]. Minerals such as copper and zinc play an important role in estrogen synthesis and metabolism by participating in the activity of various enzymes and can be supplemented with nuts and seed foods [127,128] (Table 1).

## 5. Conclusions and Perspectives

Several factors contribute to the development of KOA pain, and variations in estrogen levels are a significant contributor. Estrogen can relieve pain by regulating inflammatory responses, inhibiting cellular senescence and apoptosis, and modulating neurotransmitters. Research on osteoporosis in perimenopausal women is comparatively advanced at this time, but more research is needed to fully understand the mechanisms underlying the effect and the connection between estrogen levels and KOA pain experienced during perimenopause. Non-pharmacologic measures, as a safer and more acceptable approach, can provide some help in pain relief for perimenopausal women with KOA. Current non-pharmacological measures lack different amounts of treatment modalities for different stages of perimenopause and need to be further refined.

In addition to changes in estrogen levels, other physiologic changes in perimenopausal women, such as weight gain, loss of muscle strength, and osteoporosis, may increase the risk of developing KOA and pain. Psychological conditions in perimenopausal women also have an impact on KOA, such as increased sensitization of pain perception due to emotions such as anxiety and depression, which need to be further explored.

In conclusion, there are still a lot of unanswered questions regarding KOA pain in perimenopausal women. Future research on the mechanisms underlying this condition will be more in-depth, and more thorough and efficient treatments and preventative measures will be developed to reduce pain and enhance the quality of life for perimenopausal women with KOA.

## Figures and Tables

**Table 1:** Non-pharmacological measures for perimenopausal women with KOA.

Non-Pharmacological Measures	Types	Suggestion	Changes	Refs.
PT	AE	40 min, 5 times a week; Intensity: a moderate level of 50–70% of the maximum heart rate (220-age); walking and cycling are recommended.	Central β-endo fibular peptide↑ Estrogen↑ TNF-α↓ IL-6↓ HRV↓ Cardiovascular risk↓	[83,84,85,86]
	RT	60 min, 2–3 times a week; squats, push-ups, and hard pulls are recommended.	Estrogen↑ C reactive protein↓ TNF-α↓ IL-6↓	[87,88,89]
	MBE	60 min, 3–4 times a week; Tai Chi, Qigong, and Baduanjin are recommended.	Estrogen↑ IL-10↑ TNF-α↓ IL-6↓	[91,92,93,94,95]
PFT	LIPUS	20 min, 3–5 times a week.	GDF9↑ BMP15↑ IL-1β↓ TNF-α↓ IL-6↓	[98,99,100,101]
	TENS	20–40 min, 5 times a week; attach to specific acupoints (uterus point, Tianshu point, etc.); high-frequency TENS is more recommended.	Estrogen↑ Endorphin↑ TNF-α↓ IL-6↓ Cortisol↓	[103,104,105,106,107]
TCM	Acupuncture	20–30 min, 3–4 times a week; recommended acupoints: SP6 KI3 ST35 GB34 SP9 ST36	Estrogen↑ ER↑ IL-1β↓ TNF-α↓ IL-6↓	[108,109,110,111]
	Moxibustion	15–20 min, 3–5 times a week; recommended acupoints: CV4 SP6 KI3 ST35 GB34 SP9 ST36		[112,113,114,115]
Diet	Phytoestrogens	Recommended: soybeans soy products flaxseeds chickpeas	Estrogen↑ Osteoblast↑ Osteoclast↓	[119,120,121,122,123,124,125]
	Vitamin	Vitamin D	Estrogen↑	[126]
	Minerals	copper zinc		[127,128]

Abbreviations: CV4: Guanyuan; SP6: Sanyinjiao; KI3: Taixi; ST35: Dubi; GB34: Yanglingquan; SP9: Yinlingquan; ST36: Zusanli; E2: Estradiol; ↑: Increase; ↓: Decrease.

## Abbreviations

KOA: knee osteoarthritis; IL-1β: interleukin-1β; TNF-α: tumor necrosis factor-α; IL-6: interleukin-6; NGF: nerve growth factor; SASP: senescence-associated secretory phenotype; EVs: extracellular vesicles; MMPs: matrix metalloproteinases; VEGF: vascular endothelial growth factor; FSH: follicle-stimulating hormone; ER: estrogen receptor; ERα: estrogen receptor alpha; ROS: reactive oxygen species; BCL-2: B-cell lymphoma-2; BAX: Bcl-2-associated X protein; GPER: G protein-coupled estrogen receptor; ERβ: estrogen receptor beta; 5-HT: 5-hydroxytryptamine; GABA: gamma-aminobutyric acid; GAD: glutamate decarboxylase; PT: physical therapy; AE: aerobic exercise; HRV: heart rate variability; RT: resistance training; MBE: mind–body exercise; PFT: physical factor therapy; LIPUS: Low-Intensity Pulsed Ultrasound; TENS: Transcutaneous Electrical Nerve Stimulation; TCM: traditional Chinese medicine; GDF9: growth differentiation factor 9; BMP15: bone morphogenetic protein 15; EA: electric acupuncture; CV4: Guanyuan; SP6: Sanyinjiao; KI3: Taixi; ST35: Dubi; GB34: Yanglingquan; SP9: Yinlingquan; ST36: Zusanli; E2: Estradiol; BCA: biochanin-A.
